# Supplementary material for: IL-1-dependent enteric gliosis guides intestinal inflammation and dysmotility and modulates macrophage function
Source: Commun Biol. 2022 Aug 12;5:811. doi: 10.1038/s42003-022-03772-4 (PMC9374731; doi:10.1038/s42003-022-03772-4)
Supplement: Supplementary file 2 — Supplementary Information [file 42003_2022_3772_MOESM2_ESM.pdf]

Title: **IL-1-dependent enteric gliosis guides intestinal inflammation and dysmotility and modulates macrophage function**

Short Title: **IL-1-dependent enteric gliosis intestinal inflammation**

Authors:

Reiner Schneider[1], Patrick Leven[1], Shilpashree Mallesh[1], Mona Breßer[1], Linda Schneider[1], Elvio Mazzotta[2], Paola Fadda[2], Tim Glowka[1], Tim O. Vilz[1], Philipp Lingohr[1], Jörg C. Kalff[1], Fievos L. Christofi[2]†, Sven Wehner[1]*†

†These authors contributed equally.

Affiliations:

[1]University Hospital Bonn, Department of Surgery, Bonn, Germany.
[2]Department of Anesthesiology, Wexner Medical Center, The Ohio State University, Columbus, OH, USA.

*Corresponding author

Prof. Dr. Sven Wehner, University of Bonn, Department of Surgery, Venusberg-Campus 1, 53127 Bonn, Germany, phone: +49-228-287-11007, Sven.Wehner@ukbonn.de

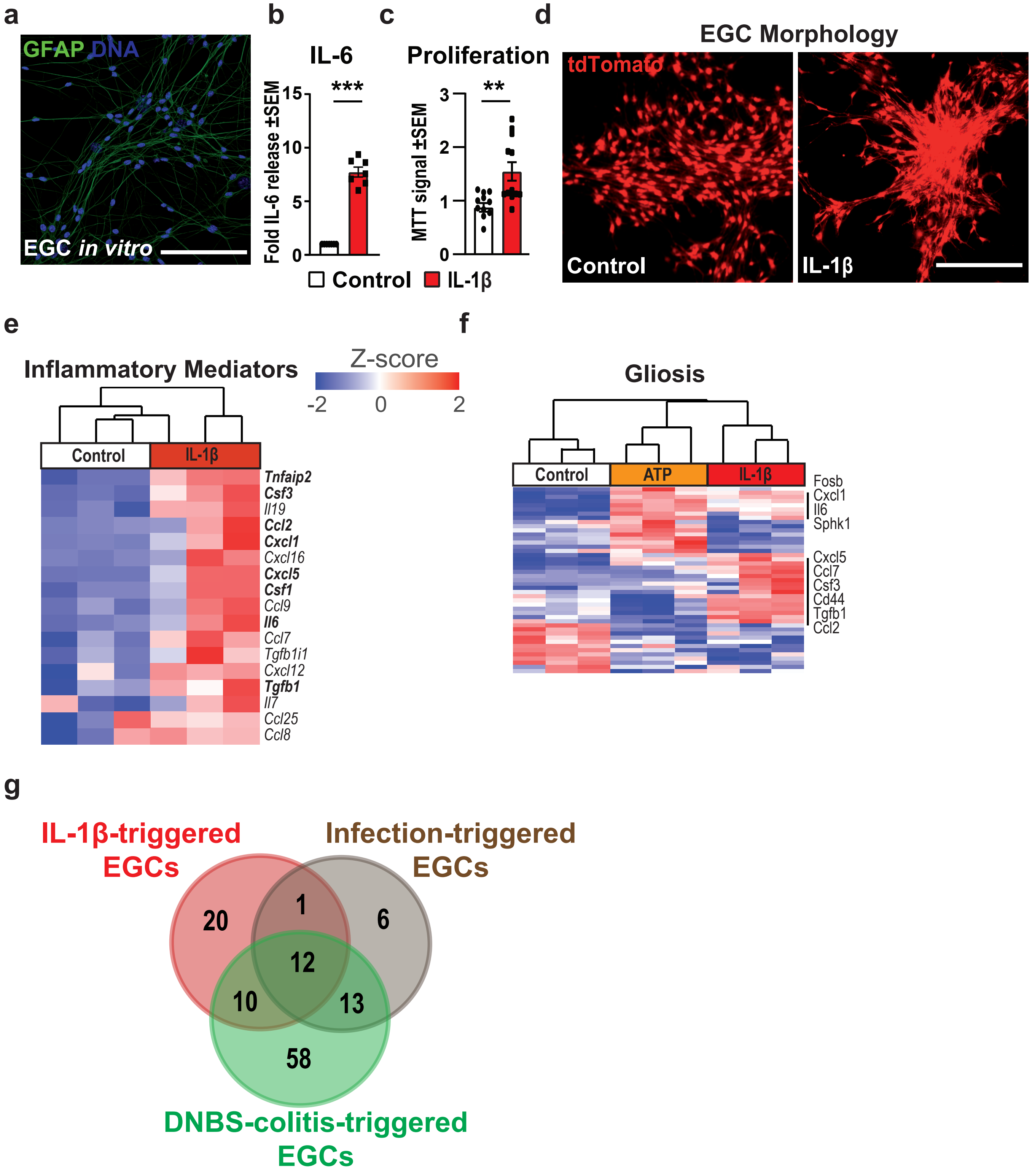

a
GFAP DNA
EGC in vitro
b
IL-6
***
Fold IL-6 release ±SEM
15
10
5
0
c
Proliferation
**
MTT signal ±SEM
3
2
1
0
Control IL-1β
d
EGC Morphology
tdTomato
Control
IL-1β
e
Inflammatory Mediators
Z-score
-2 0 2
Control
IL-1β
Tnfaip2
Csf3
Il19
Ccl2
Cxcl1
Cxcl16
Cxcl5
Csf1
Ccl9
Il6
Ccl7
Tgfb1i1
Cxcl12
Tgfb1
Il7
Ccl25
Ccl8
f
Gliosis
Control
ATP
IL-1β
Fosb
Cxcl1
Il6
Sphk1
Cxcl5
Ccl7
Csf3
Cd44
Tgfb1
Ccl2
g
IL-1β-triggered EGCs
Infection-triggered EGCs
DNBS-colitis-triggered EGCs
20
1
6
12
10
13
58

Fig. S1: IL-1 induces a specific reactive phenotype in EGCs.

(**a**) Immunocytochemistry for GFAP (green) and nuclei (Hoechst, blue) in primary EGCs. Scale bar 50 µm. (**b**) Primary EGCs were treated with IL-1β (10 ng/ml), or vehicle for 24h and processed for ELISA. n=8 per group. (**c**) Primary EGCs were treated with IL-1β (10 ng/ml) or vehicle for 24h and processed for MTT assays to investigate proliferation. n=12 per group. (**d**) Live cell imaging of primary EGCs treated with IL-1β (10 ng/ml) or vehicle for 24h. n=5. Scale bar 50µm. (**e**) Heat map of genes associated to “inflammatory mediators”. (**f**) Heat map of genes associated to “gliosis” showing EGCs treated with IL-1β (10 ng/ml), ATP (100µM) or vehicle for 24h and processed for bulk-RNA-Seq. n=3 per group. (**g**) Venn diagram of induced gliosis genes showing the mutual genes between IL-1β -triggered EGCs, infection-triggered EGCs (GSE114780) and DNBS-colitis-triggered EGCs (GSE182708). Statistics were done with Student´s t-test and Fisher´s exact test. ***<0.001, **<0.01 * were compared to untreated EGC cultures.

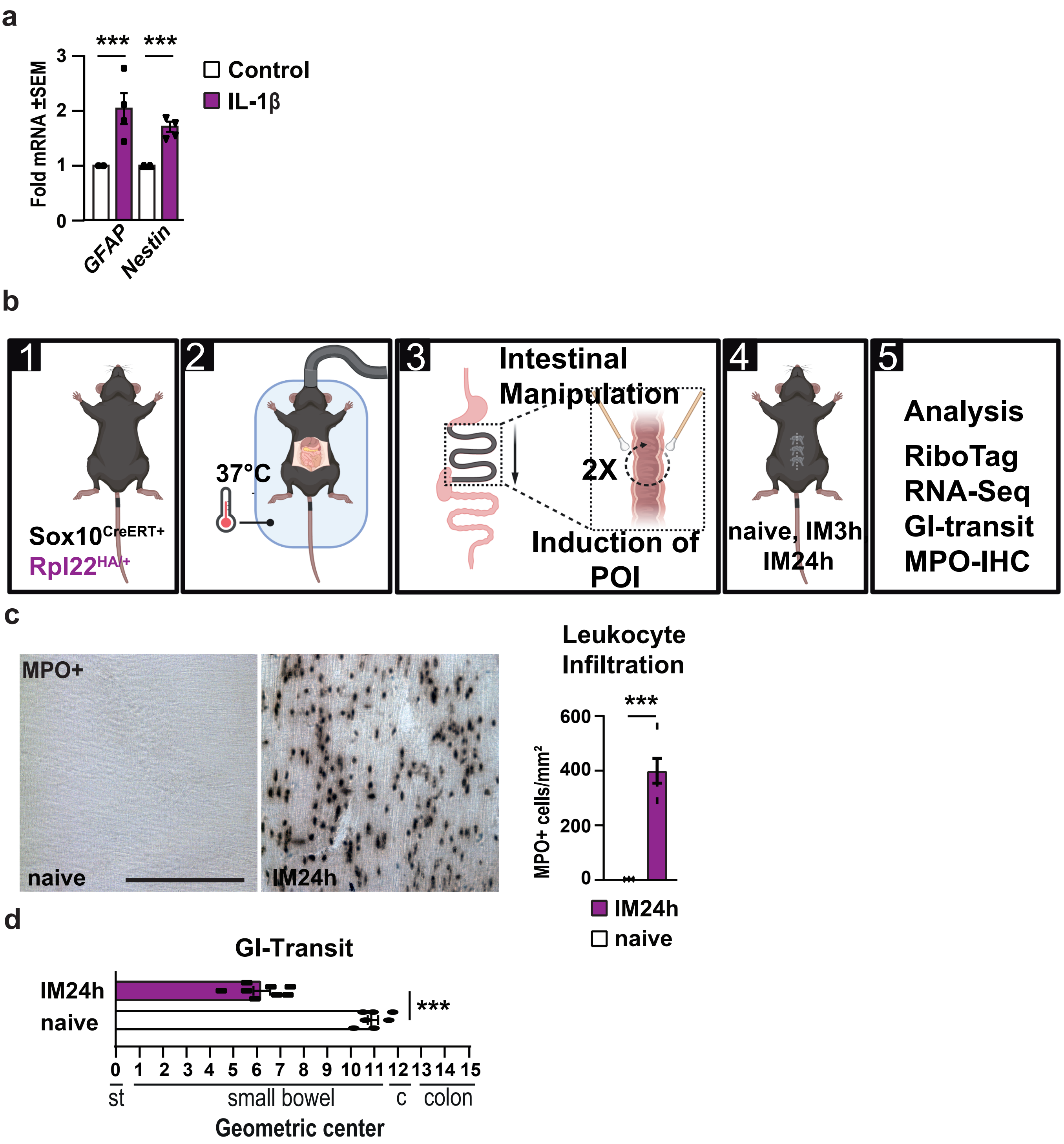
a
Fold mRNA ±SEM
***
***
Control
IL-1β
GFAP
Nestin
b
1
Sox10CreERT+
Rpl22HA/+
2
37°C
3
Intestinal Manipulation
2X
Induction of POI
4
naive, IM3h
IM24h
5
Analysis
RiboTag
RNA-Seq
GI-transit
MPO-IHC
c
MPO+
naive
IM24h
Leukocyte Infiltration
MPO+ cells/mm²
***
IM24h
naive
d
GI-Transit
IM24h
naive
***
st
small bowel
c
colon
Geometric center

Fig. S2: IL-1-induced enteric gliosis resembles the EGC reactivity profile after surgical trauma.

(**a**) qPCR analysis of EGC cultures produced from *Sox10*$^{CreERT2+}$-*Rpl22*$^{HA/+}$ mice and treated with IL-1β for 3h. Bars showing fold change compared to untreated EGCs. n=4. (**b**) Experimental scheme describing the postoperative ileus animal model, investigated disease time points, and readouts. (**c**) Histology of myeloperoxidase (MPO^{+}) cells in the *ME* of naïve and IM24h *Sox10*$^{CreERT2+}$-*Rpl22*$^{HA/+}$ animals and subsequent leukocyte infiltration quantification. n=5. Scale bar 100 µm. (**d**) GI-transit analysis in naïve and IM24h animals. n=5. Statistics were done with Student´s t-test. ***<0.001, All * compared to controls.

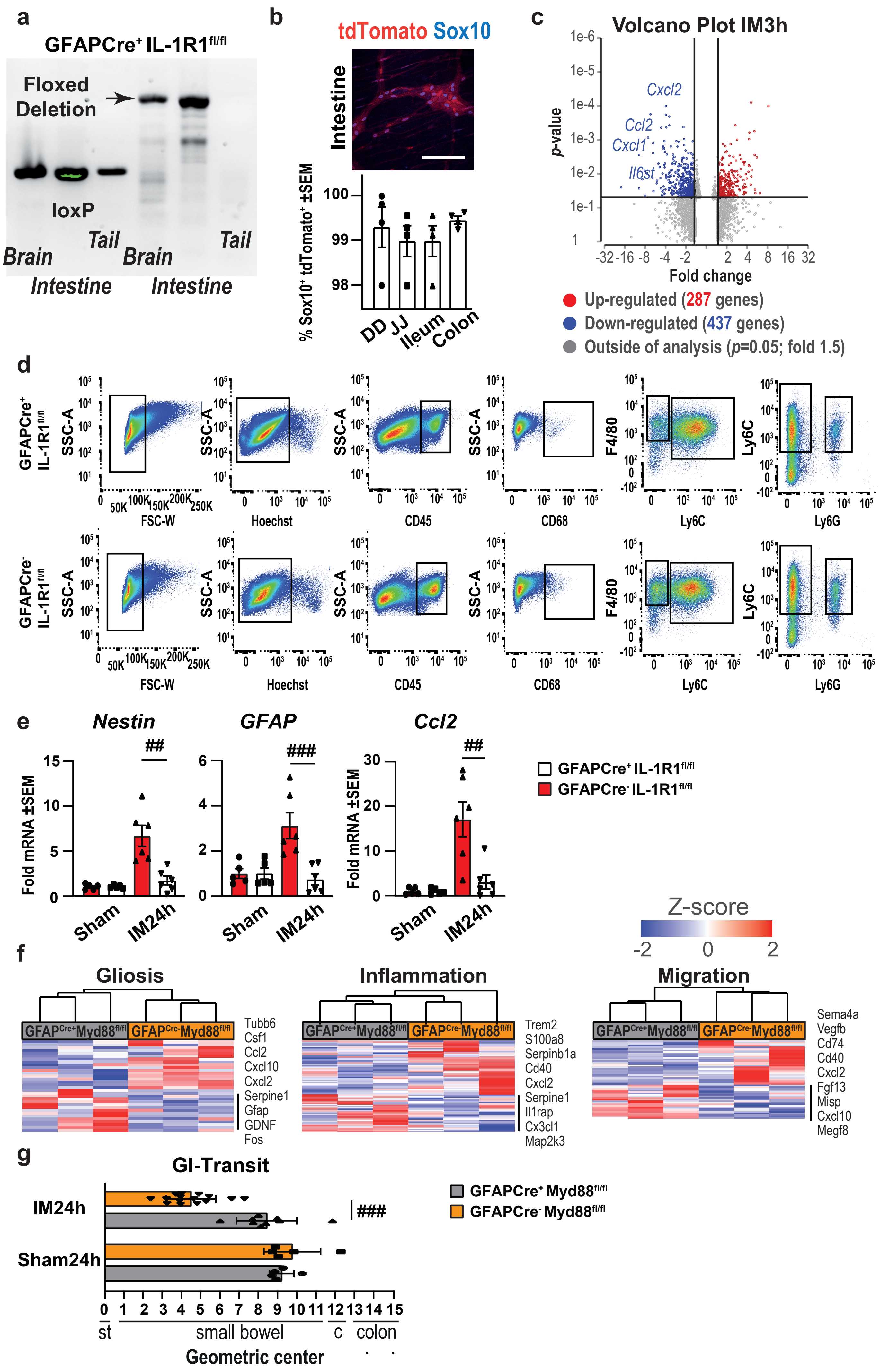
a
GFAPCre+ IL-1R1fl/fl
Floxed Deletion
loxP
Brain
Tail
Brain
Tail
Intestine
Intestine
b
tdTomato Sox10
Intestine
% Sox10+ tdTomato+ ±SEM
100
99
98
DD
JJ
Ileum
Colon
c
Volcano Plot IM3h
p-value
Fold change
Cxcl2
Ccl2
Cxcl1
Il6st
Up-regulated (287 genes)
Down-regulated (437 genes)
Outside of analysis (p=0.05; fold 1.5)
d
GFAPCre+ IL-1R1fl/fl
GFAPCre- IL-1R1fl/fl
SSC-A
FSC-W
Hoechst
CD45
CD68
F4/80
Ly6C
Ly6G
e
Nestin
GFAP
Ccl2
Fold mRNA ±SEM
##
###
##
Sham
IM24h
GFAPCre+ IL-1R1fl/fl
GFAPCre- IL-1R1fl/fl
f
Z-score
-2
0
2
Gliosis
Inflammation
Migration
GFAPCre+Myd88fl/fl
GFAPCre-Myd88fl/fl
Tubb6
Csf1
Ccl2
Cxcl10
Cxcl2
Serpine1
Gfap
GDNF
Fos
Trem2
S100a8
Serpinb1a
Cd40
Cxcl2
Serpine1
Il1rap
Cx3cl1
Map2k3
Sema4a
Vegfb
Cd74
Cd40
Cxcl2
Fgf13
Misp
Cxcl10
Megf8
g
GI-Transit
IM24h
Sham24h
###
GFAPCre+ Myd88fl/fl
GFAPCre- Myd88fl/fl
0 1 2 3 4 5 6 7 8 9 10 11 12 13 14 15
st
small bowel
c
colon
Geometric center

Fig. S3: EGC-restricted IL-1R1 deficiency prevents postoperative macrophage activation and protects mice from POI. (**a**) PCR detecting IL1R1-loxP and IL1R1-deletion sites in the brain, intestine, and tail DNA of $GFAP^{Cre+}$ and $GFAP^{Cre-}$ animals. PCR proves successful Cre-loxP deletion in the IL1R1 gene locus. (**b**) Immunohistochemistry for EGC-marker SOX10 (blue) and tdtomato (red) in the *ME* of $GFAP^{Cre+}Ai14^{fl/fl}$ mice. Quantification of double-positive cells showed a strong overlap of the $SOX10^{+}$ $tdtomato^{+}$ cells throughout the intestine (DD = duodenum, JJ = jejunum). n = 4. Scale bar 100µm. (**c**) Volcano plot showing all significantly regulated genes between Cre^{+} and Cre^{-} mice at IM3h normalized to corresponding sham groups. (**d**) $GFAP^{Cre+}$-$IL1R1^{fl/fl}$ and $GFAP^{Cre-}$-$IL1R1^{fl/fl}$ mice were analyzed at IM24h. Representative FACS analysis with gating strategy for singlets, living cells (Hoechst), CD45, CD68, Ly6C, Ly6G, F4/80 in IM24h groups of Cre^{+} and Cre^{-} mice. n=5 per group. (**e**) Gene expression analysis by qPCR for genes related to gliosis (*Nestin*, *GFAP*) and migration (*Csf1*) at IM24h in Cre^{+} and Cre^{-} mice normalized to corresponding sham groups. n=5 per group. (**f**) $GFAP^{Cre+}$-$Myd88^{fl/fl}$ and $GFAP^{Cre-}$-$Myd88^{fl/fl}$ mice underwent IM to induce POI. After 3h, RNA from the *ME* was processed for bulk-RNA-Seq analysis. Heat maps of genes involved in "gliosis", "inflammation", and "migration". n=3 per group. (**g**) $GFAP^{Cre+}$-$Myd88^{fl/fl}$ and $GFAP^{Cre-}$-$Myd88^{fl/fl}$ mice were analyzed at IM24h. GI-transit analysis in IM24h and sham groups of Cre^{+} and Cre^{-} mice. n= 5 (Sham24h); n=9-13 (IM24h). Statistics were done with Student´s t-test and Fisher´s exact test. ##<0.01, ###<0.001, All # compared to Cre- littermates.

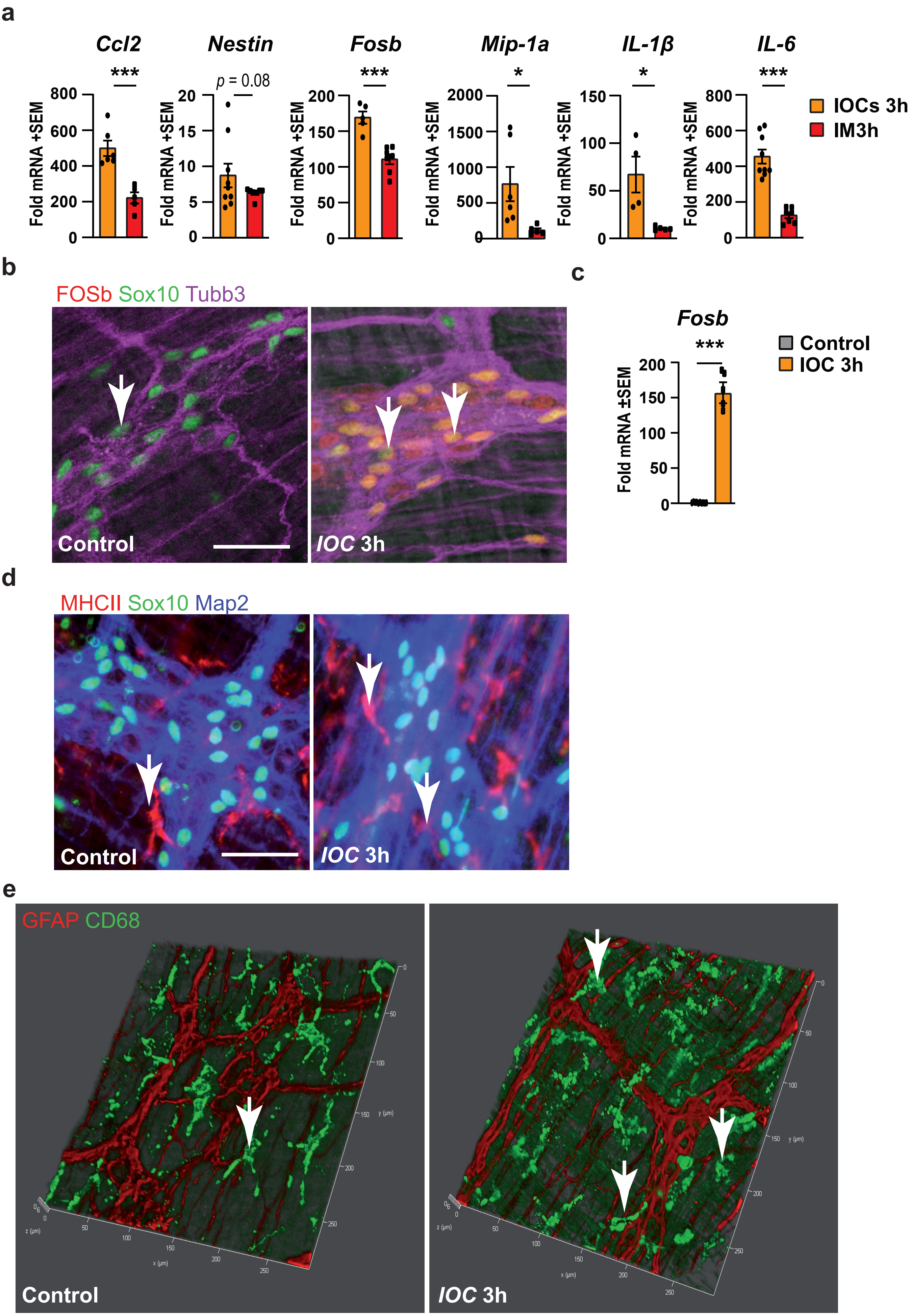

a
Ccl2
Nestin
Fosb
Mip-1a
IL-1β
IL-6
***
p = 0.08
***
*
*
***
Fold mRNA +SEM
IOCs 3h
IM3h
b
FOSb Sox10 Tubb3
Control
IOC 3h
c
Fosb
***
Fold mRNA ±SEM
Control
IOC 3h
d
MHCII Sox10 Map2
Control
IOC 3h
e
GFAP CD68
Control
IOC 3h

Fig. S4: Intestinal organotypic cultures demonstrate IL-1-dependent involvement in ME-Mac-EGC interactions.

(**a**) qPCR analysis of *ME* IOCs 3h in culture and small intestine *ME* 3h after IM for the expression of inflammatory mediators and gliosis genes. IOCs show a stronger activation of enteric gliosis and a stronger expression of chemokines and cytokines. n=4-10 (IOCs); 8-10 (IM3h). Data are shown as fold induction to untreated IOCs or naïve *ME* tissue, respectively. (**b**-**e**) Intestinal organotypic cultures (IOC) from *ME* were prepared from C57BL/6 wild-type mice jejunum by surgical dissection of the *lamina propria* and mucosa tissue, a procedure that mimics the surgical trauma *in vivo*. This model allows analysis of resident cell types in the absence of any infiltrating blood-derived immune cells. IOCs were either directly processed or incubated for 3h, corresponding to the IM3h time point in the POI mouse model. n=6 per group. (**b**) Histology for activated ($FOSb^+$, red) enteric glia ($SOX10^+$, green) and neurons ($TUBB3^+$, violet) in IOCs. White arrows mark activated EGCs ($FOSb^+$ and $SOX10^+$). Scale bar 50 µm. (**c**) Gene expression analysis by qPCR for the activation marker *Fosb*. (**d**) Histology for macrophages ($MHCII^+$, red) surrounding enteric glia ($SOX10^+$, green) and neurons ($MAP2^+$, violet) in IOCs. White arrows mark macrophages in close proximity to ganglia. Scale bar 50 µm. (**e**) Confocal z-stacks of IOCs show activated macrophages ($CD68^+$, green) surrounding EGCs ($GFAP^+$, red). Statistics were done with Student´s t-test. *<0.05, ***<0.001, All * compared to control IOCs.

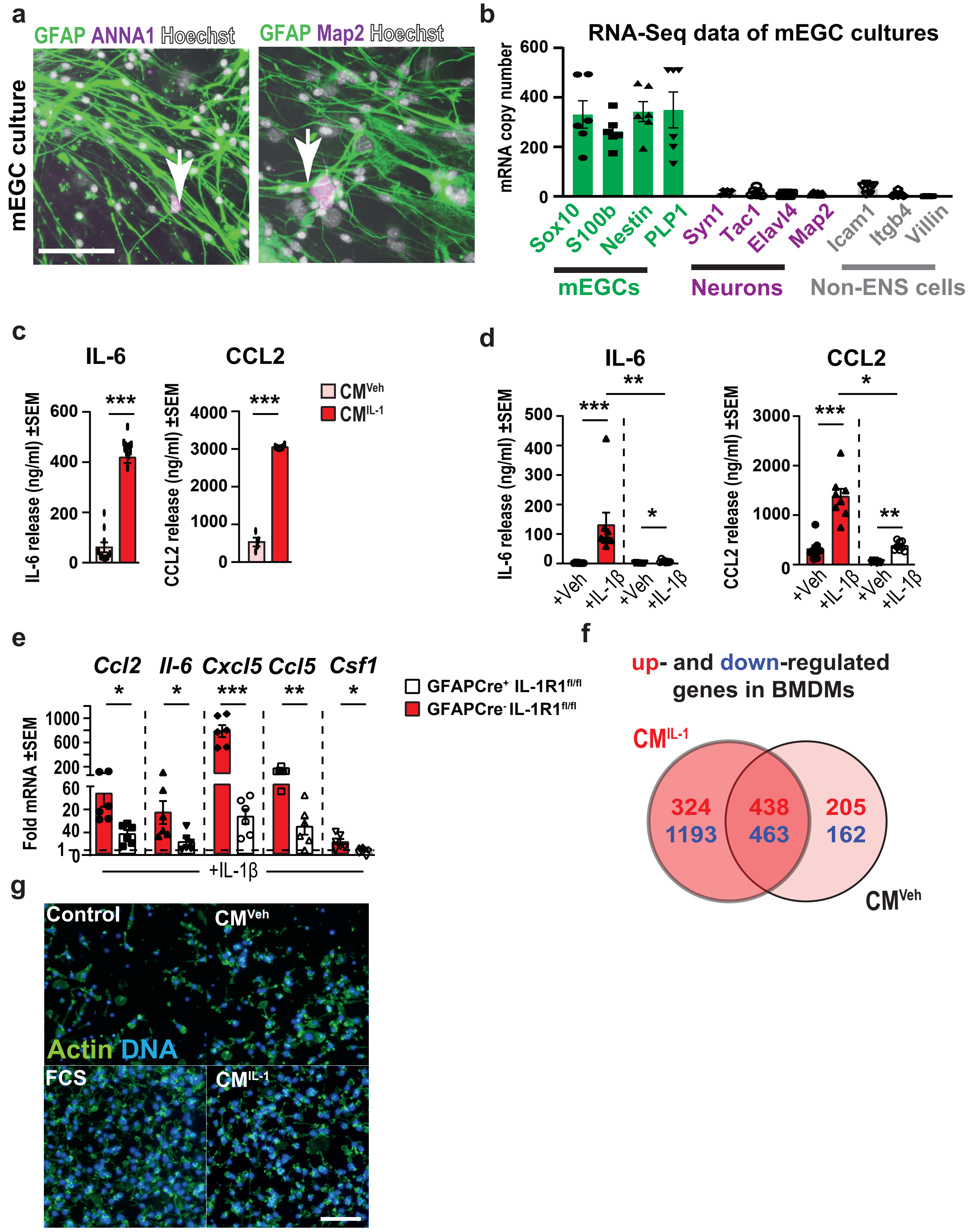
a
GFAP ANNA1 Hoechst
GFAP Map2 Hoechst
mEGC culture
b
RNA-Seq data of mEGC cultures
mRNA copy number
Sox10
S100b
Nestin
PLP1
Syn1
Tac1
Elavl4
Map2
Icam1
Itgb4
Villin
mEGCs
Neurons
Non-ENS cells
c
IL-6
CCL2
IL-6 release (ng/ml) ±SEM
CCL2 release (ng/ml) ±SEM
CMVeh
CMIL-1
d
IL-6
CCL2
+Veh
+IL-1β
e
Ccl2
Il-6
Cxcl5
Ccl5
Csf1
Fold mRNA ±SEM
+IL-1β
GFAPCre+ IL-1R1fl/fl
GFAPCre- IL-1R1fl/fl
f
up- and down-regulated genes in BMDMs
CMIL-1
324
1193
438
463
205
162
CMVeh
g
Control
CMVeh
Actin DNA
FCS
CMIL-1

Fig. S5: The impact of EGC-derived factors on macrophage function.

(**a**) Immunocytochemistry of mEGC cultures for glia (GFAP, green) and neuron (MAP2 and ANNA1, violet) markers. Hoechst (white) was used for nuclei staining. Only small numbers of enteric neurons (white arrows) were detected in mEGC cultures. Scale bar = 50µm. (b) RNA-Seq analysis for various cell type markers in EGC cultures. Only glial markers were enriched in the EGC cultures. n=6. Data is shown as RNA copies. (**c**) Primary EGCs were treated with IL-1β or vehicle for 24h. CM^{IL-1} and CM^{Veh} were investigated for IL-6 and CCL2 content by ELISA. Data is shown as ng/ml±SEM. n=10. (**d+e**) Primary EGCs isolated from $GFAP^{Cre+}$-IL1R1$^{fl/fl}$ and $GFAP^{Cre-}$-IL1R1$^{fl/fl}$ mice were treated with IL-1β or vehicle for 24h. (d) Media from both cultures were investigated for IL-6 and CCL2 content by ELISA. Cre^{+}-EGC cultures show less production of the inflammatory mediators IL-6 and CCL2. n = 3 independent EGC cultures with 2-3 technical replicates per meassurments. Data is shown as ng/ml ±SEM. (e) qPCR analysis for chemokines and cytokines produced from primary EGCs after IL-1β treatment. Cre^{+}-EGC cultures show less activation and less production of inflammatory mediators. Data are shown as fold induction ±SEM. n = 6. (**f**) Bone-marrow-derived macrophages (BMDMs) were isolated from IL1R1-KO mice to exclude any side effects from IL1β residues in the collected CM^{IL-1}. After BMDM maturation, cells were treated with CM^{IL-1}, CM^{Veh}, or left untreated for 3 and 24h. Venn diagram of significantly up-and down-regulated genes (Fold 1.5, p-value 0.05) shared by CM^{IL-1} or CM^{Veh}-treated BMDM groups. (**g**) Immunocytochemistry for actin (green) and nuclei (Hoechst, blue) of trans-migrating cells after CM^{IL-1}, CM^{Veh}, and FCS (10%) stimulation. n=4 per group. Scale bar 50 µm. Statistics were done with Student´s t-test and Fisher´s exact test. *<0.05, **<0.01, ***<0.001, All * compared to control EGCs.

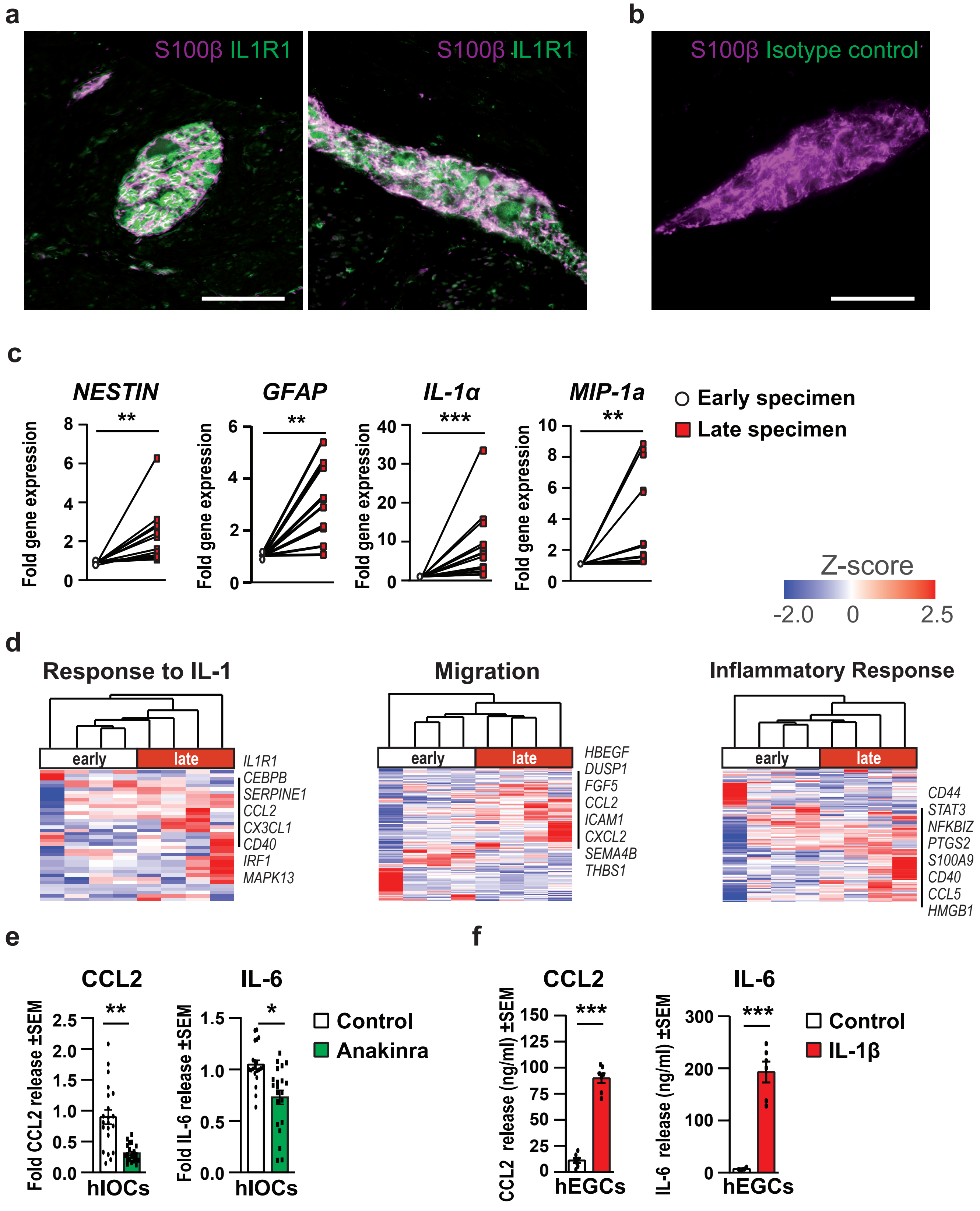
a
S100β IL1R1
S100β IL1R1
b
S100β Isotype control
c
NESTIN
GFAP
IL-1α
MIP-1a
**
**
***
**
Fold gene expression
Early specimen
Late specimen
Z-score
-2.0
0
2.5
d
Response to IL-1
Migration
Inflammatory Response
early
late
IL1R1
CEBPB
SERPINE1
CCL2
CX3CL1
CD40
IRF1
MAPK13
HBEGF
DUSP1
FGF5
CCL2
ICAM1
CXCL2
SEMA4B
THBS1
CD44
STAT3
NFKBIZ
PTGS2
S100A9
CD40
CCL5
HMGB1
e
CCL2
IL-6
Fold CCL2 release ±SEM
Fold IL-6 release ±SEM
hIOCs
Control
Anakinra
f
CCL2
IL-6
CCL2 release (ng/ml) ±SEM
IL-6 release (ng/ml) ±SEM
hEGCs
Control
IL-1β

Fig. S6: Enteric gliosis and IL-1 signaling are involved in acute intestinal inflammation after abdominal surgery.
(**a**) IL1R1 immunoreactivity in EGCs of the myenteric plexus in the human intestine. IL1R1 (red) co-labeling occurs in S100β^{+} EGCs (violet). Scale bar 100 µm. (**b**) Isotype control for IL1R1 antibody with S100β^{+} EGCs (violet). Scale bar 100 µm. (**c**) Human jejunal *ME* specimens were collected at an early and late time point during pancreatic head resection. qPCR analysis of jejunal *ME* specimens for NESTIN, GFAP, IL-1α, and MIP-1a. n=11-13; NESTIN (13), GFAP (12), IL-1α (11), MIP-1a (13). (**d**) Heat maps of genes involved in "response to IL-1", "migration", and "inflammatory response" compared between early and late surgical specimens. (**e**) Human IOCs were isolated from jejunal patient samples collected at a late time point during pancreatic head resection and incubated for 24h with Anakinra (100µg/ml) or control. Media from both cultures were investigated for IL-6 and CCL2 content by ELISA. Anakinra treated human IOCs show less production of the inflammatory mediators IL-6 and CCL2. n = 16. (**f**) ELISA analyses for CCL2 and IL-6 of primary human EGCs treated with IL-1β (0.3 ng/ml) or vehicle for 24h. n= 10. Statistics were done with Student´s t-test and Fisher´s exact test. *<0.05, **<0.01, ***<0.001, All * compared to early ME specimens or untreated hEGCs.

Supplementary tables

Supplementary Table S1: Human subjects and GI surgical specimens for IHC and hEGC cultures

#	Patient ID	Sex	Age	Surgical procedure	GI region
**1**	**PSG154**	male	64	Colectomy partial	*sigmoid colon*
**2**	**PSG270**	male	64	Colectomy partial, open	*sigmoid colon*
**3**	**PSG271**	male	50	Sigmoidectomy, laparoscopic	*sigmoid colon*
**4**	**PSG287**	female	52	Colectomy partial, laparoscopic	*ascending colon*
**5**	**PSG288**	male	68	Colectomy partial, open	*sigmoid colon*
**6**	**PSG291**	female	56	Colectomy partial, laparoscopic	*descending colon*
**7**	**PSG294**	female	66	Colectomy partial, open	*sigmoid colon*
**8**	**ETBRPOI-002**	female	43	Roux-en-Y	*jejunum*
**9**	**ETBRPOI-003**	female	34	Roux-en-Y	*jejunum*
**10**	**ETBRPOI-004**	female	42	Roux-en-Y	*jejunum*

Supplementary Table S1 summarizes information on the 14 human subjects that gave written consent to procure gut surgical tissue for *in vitro* studies. Tissue was used to isolate ganglia, prepare hEGC cultures for study, or paraffin sections for immunofluorescence staining (including jejunum IL1R1-IHC and qPCR analysis). Studies were conducted under IRB protocols.

Supplementary Table S2: Human subjects and GI surgical specimens for analysis of gliosis

#	Patient ID	Sex	Age	Start Time of procedure	Collection time points	Previous illness	Medications
**1**	160303_hME	male	55	*09:18*	*11:35 and 14:00*	**Diabetis mellitis type II**	Janumet (diabetis)<br>**Ramipril** (ACE inhibitor)<br>Cedur (blood lipids reducer)<br>**Pantozol** (gastric acid inhibitor)
**2**	160331_hME	male	85	*08:50*	*10:50 and 12:16*	none	Ursofalk (gallstone reducer)<br>Simavastin (Chlosterol reducer)<br>**Pantozol**<br>**Clexane** (coagulation inhibitor)
**3**	160405_hME	male	79	*09:14*	*11:15 and 12:40*	**art. hypertension**<br>polyneuropathy	**Pantozol**<br>**Ramipril**<br>**Alna** (prostate gland hyperplasia)<br>**Clexane**<br>Pregabador (neuropathy)<br>Riopan (gastric acid inhibitor)
**4**	180220_hME	male	80	*8:30*	*12:20 and 13:10*	benign hyperplasia of the prostate gland<br>**art. hypertension**<br>cardiac arrhythmia<br>duodenal ulcer	**Pantazol**<br>Propafenone HCl (cardiac arrhythmia)<br>**Alna**<br>Valsartan/<br>Metohexal (antihypertensive drugs)
**5**	180221_hME	male	74	*10:00*	*13:45 and 14:15*	benign hyperplasia of the prostate gland<br>**art. hypertension**<br>hyperlipidemia	**Alna**<br>Simvastatin<br>**Pantozol**<br>Beloc (antihypertensive drug)<br>ASS<br>Kalinor (potassium donor)<br>Xarelto (coagulation inhibitor)
**6**	180424 hME	female	61	*08:30*	*11:35 and 13:50*	moderate aorta insufficiency<br>rheumatoid arthritis<br>reflux esophagitis<br>**art. hypertension**<br>hypercholesterolemi a	Bisoprolol (ß-blocker)<br>Euthyrox (L-Thyroxin)<br>Lantarel (arthritis)<br>Lisinopril (blood pressure reducer)<br>Rantudil (arthritis)<br>Methothrexat/Golimumab (arthritis)
**7**	180821_hME	male	59	*9:00*	*12:15 and 13:35*	hyperplasia of the prostate gland<br>sleep apnea syndrome<br>kidney cyst right side	Amlodipin (antihypertensive drug)<br>**Pantozol**
**8**	181029_hME	female	73	*07:45*	*10:30 and 11:15*	COPD<br>Myxoma<br>**Diabetis mellitus Type II**<br>**hypertension**<br>hyperlipidemia	Berotec (COPD)<br>Inuvair (COPD)<br>Spiriva (COPD)<br>**clexane**<br>Bisoprolol (blood pressure reducer)<br>**Ramipril**<br>HCT (diuretic drug)<br>Marcumar<br>**Atorvastatin**

**9**	190429_hME	female	61	*9:15*	*13:58 and 15:13*	**Diabetis mellitus**<br>**art. hypertension**	**Pantazol**<br>Amlodipin
**10**	190506_hME	male	76	*8:55*	*12:12 and 13:00*	Ulcera Duodeni<br>reflux esophagitis II°<br>sigma diverticulosis<br>**Diabetis mellitus Type II**	ASS<br>**Atorvastatin** (cholesterol reducer)<br>Gabapentin (antiepilepthic)<br>Amitriptilin (antidepressant)
**11**	190805_hME	male	29	*10:24*	*12:00 and 14:29*	pancreas insufficiency,<br>reflux esophagitis,<br>opticus neuropathy,<br>ambiguous tremor,<br>benigne hyperplasia of prostate gland,<br>depression	Clonazepam/Propranolol (tremor)<br>Mitrazapin/Risperdal/Anafranil (antidepressants)<br>Mictinorm (control of bladder function)
**12**	190806_hME	female	30	*10:01*	*12:25 and 13:30*	von Hippel-Lindau syndrome<br>pheochromocytoma	Paracetamol<br>**Clexane**
**13**	190808_hME	female	70	*10:00*	*13:40 and 18:20*	**pul./art. hypertension**<br>mitral-/tricuspidal regurgitation<br>chron. pancreatitis<br>sec. **Diabetis mellitus Type 3c**<br>kidney insufficiency (dialysis)<br>vein thrombosis	Marcumar (antikoagulans)<br>**Atorvastatin**<br>**Pantozol**<br>Bisopropol (ß-blocker)<br>Torem (urine excretion)<br>Amitriptylin
**14**	190917_hME	female	51	*9:21*	*12:07 and 12:55*	none	**Pantozol**<br>Sertralin (antidepressant)<br>Paracetamol<br>**Clexane**
**15**	191017_hME	male	75	*9:11*	*13:38 and 15:22*	silicosis<br>gastritis	**Pantozol**<br>Eliquis (coagulation inhibitor)<br>**Alna**
**16**	191023_hME	male	57	*9:11*	*11:20 and 14:10*	cholestasis<br>acute pancreatitis<br>NSTEMI (myocardial infarction)<br>**art. hypertension**<br>obesity<br>**Diabetis mellitus Type II**<br>hyperlipidemia	Levemir (insulin)<br>ASS<br>Amlodipin<br>Metformin (antidiabetic)<br>**Pantozol**<br>Rekawan (potassium donor)<br>Simvastin (cholesteol reducer)
**17**	191119_hME	male	62	*9:04*	*11:20 and 12:20*	aortic aneurysm	**Clexane**
**18**	200109_hME	male	64	*9:18*	*12:10 and 13:25*	kidney cell carcinoma<br>hypercholesterolemia<br>**art. hypertension**<br>**Diabetis mellitus Type II**<br>nicotine abuse	Telmisartan (blood presure reducer)<br>Metformin<br>**Atorvastatin**<br>**Pantoprazole**<br>Actrapid (insulin)<br>Lantus (insulin)
**19**	200326_hME	female	71	*9:28*	*13:22 and 14:42*	peripheral arterial disease	ASS<br>Allopurinol (uric acid reducer)

						constriction of inner carotis<br>**art. hypertension**	Bisohexal/Candesartanicilex etil (blood pressure reducer)<br>**Pantozol**
**20**	200729_hME	female	78	*9:27*	*10:55 and 11:45*	hyperthyroidism<br>carpal tunnel syndrome	**Pantozo**l<br>L-Thyroxin
**21**	200811_hME	female	68	*8:55*	*10:40 and 12:25*	lung carcinoma<br>jaw angle carcinoma<br>**art. hypertension**<br>**Diabetes mellitus**	**Pantozol**<br>L-Thyroxin<br>Metoprolol (blood pressure reducer)<br>**Ramipril**<br>Amlodipin<br>Mirtazapine<br>Xipamid (urine excretion)<br>Metform (blood sugar reducer)<br>Kalinor<br>**Clexane**
**22**	200818_hME	female	62	*9:30*	*11:18 and 12:45*	**art. hypertension**<br>obesity<br>chron. pain syndrome<br>hypothyroidism	Riopan<br>**Clexane**<br>**Ramipril**<br>**Pantozol**<br>L-Thyroxin<br>Levemir<br>Metformin<br>Actrapid

Supplementary Table S2: Information on 16 patients who underwent pylorus-preserving pancreaticoduodenectomy and gave written consent to procure jejunal tissue for qPCR studies. The tissue was used for RNA isolation, cDNA preparation, and gene expression analysis. The study was conducted under protocols permitted by the ethical committee of the University of Bonn, Germany.

Supplementary Table S3: Oligonucleotide primers

Gene	Forward	Reverse
mIL-1β	TTGACGGACCCCAAAAGATG	CAGGACAGCCCAGGTCAAAG
mCD68	TGACCTGCTCTCTCTAAGGCTACAG	AGGACCAGGCCAATGATGAG
mMip-1a	ACCATGACACTCTGCAACCA	CCCAGGTCTCTTTGGAGTCA
mCsf-1	GTGTCAGAACACTGTAGCCAC	TCAAAGGCAATCTGGCATGAAG
mTnfα	CTGAACTTCGGGGTGATCGG	GGCTTGTCACTCGAATTTTGAGA
mGFAP	ACATCGAGATCGCCACCTAC	CCTTCTGACACGGATTTGGT
mNestin	AGATCGCTCAGATCCTGGAA	AGGTGTCTGCAAGCGAGAGT
mIL-6	AAGTCGGAGGCTTAATTACACATGT	CCATTGCACAACTCTTTTCTCATT
mFosb	ATGGGCTCTCCTGTCAACAC	ACGGAGGAGACCAGAGTGG
mCcl2	CCCAATGAGTAGGCTGGAGA	GCTGAAGACCTTAGGGCAGA
mCcl5	GTGCCCACGTCAAGGAGTAT	CCCACTTCTTCTCTGGGTTG
mCxcl2	TCCAGGTCAGTTAGCCTTGC	CGGTCAAAAAGTTTGCCTTG
mCxcl5	CGCTAATTTGGAGGTGATCC	AACACTGGCCGTTCTTTCC
18S-RNA	GTGGAGCGATTTGTCTGGTT	ACGCTGAGCCAGTCAGTGTA
18S-RNA	Assay ID 4319413E	
hIL-1α	TGGTAGTAGCAACCAACGGGA	ACTTTGATTGAGGGCGTCATTC
hIL-1β	AGCTACGAATCTCCGACCAC	CGTTATCCCATGTGTCGAAGAA
hGFAP	CTGCGGCTCGATCAACTCA	TCCAGCGACTCAATCTTCCTC
hNESTIN	GGGAGTTCTCAGCCTCCAG	GGAGAAACAGGGCCTACAGA
hCCL2	ATAACAGCAGGTGACTGGGG	CCAGCATGAAAGTCTCTGCC
hMIP-1a	GCTGACTACTTTGAGACGAGC	CCAGTCCATAGAAGAGGTAGC
hIL-6	GTCAGGGGTGGTTATTGCAT	AGTGAGGAACAAGCCAGAGC
hCXCL2	GGCAGAAAGCTTGTCTCAACCC	CTCCTTCAGGAACAGCCACCAA

Supplementary Table S3 summarizes information on all used qPCR primers.

Supplementary Table S4: Antibodies used in the study

Target	Dye/Dilution	Secondary antibody*	Use	Company
CD45	Pacific blue / 1:200		FACS	Biolegend Clone: 30-F11 #103126
Ly6C	APC / 1:300		FACS	eBioscience Clone: HK1.4 # 17-5932-82
CD68	Alexa647 / 1:400		FACS	Biolegend Clone: FA-11 #137003
Ly6G	FITC / 1:300		FACS	Biolegend Clone: 1A8 #127605
F4/80	PE / 1:400		FACS	Biolegend Clone: BM8 #123110
MHCII	1:400	AlexaFluor647 or Cy3	IHC	BioLegend Clone:M5/114.15.2 # 107602
GFAP	1:1000	AlexaFluor647	IHC/ICC	Synaptic System # 173002
Sox10	1:400	Cy3	IHC/ICC	Santa Cruz # sc- 17342
FOSb	1:200	Cy3	IHC	Santa Cruz # sc52
s100β	1:500	Cy3	IHC	Sigma S2532
IL1R1	1:100	Cy2	IHC	Abcam # ab106278
HA-Tag (C29F4)	1:500	Cy5	IHC	cell signalling #3724
CD68	1:500	Cy2	IHC	Biorad # MCA1957
Phalloidin		Alexa488-coupled		Life Technologies #A12379
CD68	1:400	Cy2	IHC	Biolegend #333802
GFAP	1:1000	Cy3	IHC	DAKO #Z 0334
Map2	1:2000	Cy2	IHC	Biolegend #BLD-822501
Tubb3	1:1000	Cy5	IHC	Biolegend #801201
Hoechst	1:5000		ICC/IHC	Thermo Scientific #H3570

Supplementary Table S4 summarizes information on all used antibodies.

Supplementary Table S5: Overlap of genes between IL-1-triggered EGCs, infection-triggered EGC, and DNBS-colitis-triggered EGCs

Gene Names
Cd44
Tubb6
Tnc
Ccl7
Tnfrsf12a
Hmox1
Lgals1
Vcan
Ccl2
Timp1
Serpina3n
Lgals3

Supplementary Table S5 summarizes information on the RNA-Seq analysis of EGCs triggered with IL-1β and infection and DNBS-colitis.

Supplementary Table S6: Overlap of genes between IL-1-triggered EGCs and IM3h-RiboTag-EGCs

Gene name
Ptgs2
Cd44
Csf1
Gcnt2
Tgfb1
Tubb6
Icam1
Tnc
Tnfrsf12a
Rfc3
Cp
Hmox1
Epha4
Pvr
Ptx3
Vcan
Ccl2
Ctsb
Thbs1
Srxn1
Cxcl1
Sphk1
Timp1
Hmga1
Il6
Cxcl2

Supplementary Table S6 summarizes information on the RNA-Seq analysis of EGCs triggered with IL-1β and glial gene expression from *RiboTag* mice at IM3h.
