# Supplementary material for: IL-1-dependent enteric gliosis guides intestinal inflammation and dysmotility and modulates macrophage function
Source: Commun Biol. 2022 Aug 12;5:811. doi: 10.1038/s42003-022-03772-4 (PMC9374731; doi:10.1038/s42003-022-03772-4)
Supplement: Supplementary file 3 — Description of Additional Supplementary Files [file 42003_2022_3772_MOESM3_ESM.pdf]

Description of Additional Supplementary Files

File name: Supplementary Data 1
Description: Raw data used for the main figures.
