# Supplementary material for: IL-1-dependent enteric gliosis guides intestinal inflammation and dysmotility and modulates macrophage function
Source: Commun Biol. 2022 Aug 12;5:811. doi: 10.1038/s42003-022-03772-4 (PMC9374731; doi:10.1038/s42003-022-03772-4)
Supplement: Supplementary file 5 — Reporting Summary [file 42003_2022_3772_MOESM5_ESM.pdf]

nature portfolio

Corresponding author(s): COMMSBIO-21-3484-T

Last updated by author(s): Jun 13, 2022

Reporting Summary

Nature Portfolio wishes to improve the reproducibility of the work that we publish. This form provides structure for consistency and transparency in reporting. For further information on Nature Portfolio policies, see our Editorial Policies and the Editorial Policy Checklist.

Statistics

For all statistical analyses, confirm that the following items are present in the figure legend, table legend, main text, or Methods section.

n/a	Confirmed	
☐	☒	The exact sample size (*n*) for each experimental group/condition, given as a discrete number and unit of measurement
☐	☒	A statement on whether measurements were taken from distinct samples or whether the same sample was measured repeatedly
☐	☒	The statistical test(s) used AND whether they are one- or two-sided<br>*Only common tests should be described solely by name; describe more complex techniques in the Methods section.*
☐	☒	A description of all covariates tested
☒	☐	A description of any assumptions or corrections, such as tests of normality and adjustment for multiple comparisons
☒	☐	A full description of the statistical parameters including central tendency (e.g. means) or other basic estimates (e.g. regression coefficient) AND variation (e.g. standard deviation) or associated estimates of uncertainty (e.g. confidence intervals)
☐	☒	For null hypothesis testing, the test statistic (e.g. *F*, *t*, *r*) with confidence intervals, effect sizes, degrees of freedom and *P* value noted<br>*Give P values as exact values whenever suitable.*
☒	☐	For Bayesian analysis, information on the choice of priors and Markov chain Monte Carlo settings
☒	☐	For hierarchical and complex designs, identification of the appropriate level for tests and full reporting of outcomes
☒	☐	Estimates of effect sizes (e.g. Cohen's *d*, Pearson's *r*), indicating how they were calculated

Our web collection on statistics for biologists contains articles on many of the points above.

Software and code

Policy information about availability of computer code

Data collection	n/a
Data analysis	n/a

For manuscripts utilizing custom algorithms or software that are central to the research but not yet described in published literature, software must be made available to editors and reviewers. We strongly encourage code deposition in a community repository (e.g. GitHub). See the Nature Portfolio guidelines for submitting code & software for further information.

Data

Policy information about availability of data

All manuscripts must include a data availability statement. This statement should provide the following information, where applicable:

- Accession codes, unique identifiers, or web links for publicly available datasets
- A description of any restrictions on data availability
- For clinical datasets or third party data, please ensure that the statement adheres to our policy

The datasets produced in this study are available upon reasonable request and in the following databases: RNA-Seq data from mRNA of ME-tissue from patients in GSE149181 accessible here: https://www.ncbi.nlm.nih.gov/geo/query/acc.cgi?acc=GSE149181.
The RiboTag data of control and IM3h mice is available under the accession number GSE198889 (token: cdupiqaotjsnvgf), and the data of EGCs treated with IL1β, and BMDMs treated with conditioned media is available under the accession number GSE205610 (token: qlmrwskulxyfpip).

Field-specific reporting

Please select the one below that is the best fit for your research. If you are not sure, read the appropriate sections before making your selection.

☒ Life sciences ☐ Behavioural & social sciences ☐ Ecological, evolutionary & environmental sciences

For a reference copy of the document with all sections, see nature.com/documents/nr-reporting-summary-flat.pdf

Life sciences study design

All studies must disclose on these points even when the disclosure is negative.

Sample size	For animal experiments, all calculations were done in advanced to get the govermental approval for the studies.
Data exclusions	n/a
Replication	n/a
Randomization	Animal genotypes were not known by the scientist performing the experiments.
Blinding	Animal genotypes were not known by the scientist performing the experiments.

Reporting for specific materials, systems and methods

We require information from authors about some types of materials, experimental systems and methods used in many studies. Here, indicate whether each material, system or method listed is relevant to your study. If you are not sure if a list item applies to your research, read the appropriate section before selecting a response.

Materials & experimental systems

n/a	Involved in the study	
☐	☒	Antibodies
☒	☐	Eukaryotic cell lines
☒	☐	Palaeontology and archaeology
☐	☒	Animals and other organisms
☐	☒	Human research participants
☒	☐	Clinical data
☒	☐	Dual use research of concern

Methods

n/a	Involved in the study	
☒	☐	ChIP-seq
☐	☒	Flow cytometry
☒	☐	MRI-based neuroimaging

Antibodies

Antibodies used	See the supplementary table 3
Validation	done by the companies.

Animals and other organisms

Policy information about studies involving animals; ARRIVE guidelines recommended for reporting animal research

Laboratory animals	Experiments were performed using 8-12-week-old Sox10CreERT2xRpl22HA/+ or GFAPcrexIL1R1fl/fl mice kept in a pathogen-free animal facility with standard rodent food and tap water ad libitum. Appropriate authorities of North-Rhine-Westphalia, Germany (81-02.04.2016.A367) approved experiments.
Wild animals	n/a
Field-collected samples	n/a
Ethics oversight	n/a

Note that full information on the approval of the study protocol must also be provided in the manuscript.

Human research participants

Policy information about studies involving human research participants

Population characteristics	n/a
Recruitment	n/a
Ethics oversight	n/a

Note that full information on the approval of the study protocol must also be provided in the manuscript.

Flow Cytometry

Plots

Confirm that:

☒ The axis labels state the marker and fluorochrome used (e.g. CD4-FITC).

☒ The axis scales are clearly visible. Include numbers along axes only for bottom left plot of group (a 'group' is an analysis of identical markers).

☒ All plots are contour plots with outliers or pseudocolor plots.

☒ A numerical value for number of cells or percentage (with statistics) is provided.

Methodology

Sample preparation	Isolation of ME was achieved by sliding small bowel segments onto a glass rod, removing the outer muscularis circumferentially with moist cotton applicators and cutting the ME into fine pieces. ME was digested with a 0.1% collagenase type II (Worthington Biochemical, Lakewood, NJ, USA) enzyme mixture, diluted in PBS, containing 0.1 mg/ml DNase I (La Roche, Germany), 2.4 mg/ml Dispase II (La Roche, Germany), 1 mg/ml BSA (Applichem), and 0.7 mg/ml trypsin inhibitor (Applichem) for 40 min in a 37°C shaking water bath. Afterwards single cell suspension was obtained using a 70 µm filter mesh and cells were stained for 30 min at 4°C with the appropriate antibodies. For antibodies used in this study see supplementary table S4. Flow cytometry analyses were performed on FACSCanto I (BD Biosciences) using FACSDiva software and data were analyzed with the latest FlowJo software (Tree Star, Ashland, OR, USA).
Instrument	FACS Canto I
Software	Flow Jo and FACSDiva
Cell population abundance	CD45 was the main pre-gate.
Gating strategy	Described in the suplementary.

☒ Tick this box to confirm that a figure exemplifying the gating strategy is provided in the Supplementary Information.
